# Mr. Shaw, on Hydrocephalus Internus

**Published:** 1800-06

**Authors:** J. T. Shaw

**Affiliations:** Dudley, Worcestershire


### *To the Editors of the Medical and Phyſical Journal.*

Gentlemen,

If you think the following caſes of Hydrocephalus Internus worthy a place in your valuable Journal, by inſerting them in your next Number you will much oblige,

Gentlemen,

Your obedient humble ſervant,

J. T. SHAW.

*Dudley, Worceſterſhire,*
*April* 11, 1800.

---

—— Timmin's ſon, ætat. 6, of an healthful and ſanguine temperament, had for ſome time paſt been obſerved by his parents to have had worms, from the voracіouſneſs of his appetite and and the unuſual ſize of his belly, as likewiſe from his having voided ſeveral at different times; but as his health, in other reſpects, was not affected, they did not think it neceſſary to apply for any advice.

*March* 1. For a week paſt he has complained of a cold ſhivering, heat, &c. which continues with other ſymptoms of pyrexia; he had a ſaline mixture with an antimonial powder, which operated as an emetic, and brought off a large quantity of bile from his ſtomach with evident relief.

*March* 2. To-day I found him in extreme agony, ſcreaming and ſtruggling in a violent degree; at intervals he lies in a comatoſe ſtate, taking no kind of notice of any one, and if diſturbed again, recurring into the ſcreaming paroxyſms; p. 100, ſmall and weak, belly ſoft, of its natural ſize; had a looſe ſtool yeſterday after the operation of the emetic; tongue parched, ſkin hot and dry; drinks whatever is given him, but at times ſeems to ſwallow with difficulty; will not take any thing in ſubſtance, but refuſes not panada, gruel, &c. The pupils are dilated, the left rather more than the right, and they do not contract on expoſure to a ſudden and ſtrong light. I had his head ſhaved and bathed with vinegar; the apparent eaſe it gave, made me perſevere in its uſe for ſome time, till its effects ceaſed; a bliſter was applied to the vertex, and iij grs. of calomel ordered twice a day. Ten o'clock, P. M. p. 96, fuller and rather harder; ſtill continues in the ſame ſtate, ſcreaming and toſſing his head about, unleſs held by the attendants, and will not be put off the lap: although we cannot get him to ſpeak, at intervals he ſeems to be ſenſible.

*March* 3. P. 100, weak and ſoft; has had no ſtool; belly ſtill ſoft; paſſed a reſtleſs and diſturbed night, ſeemingly in great agony, moaning during the ſhort intervals he was free from the ſcreaming paroxyſms; makes but little water, and that involuntary; takes whatever fluid is given him. Cont. miſt. ſaline et calomel.

*March* 4. P. 120, belly ſoft, and no ſtool; ſkin dry; has made more urine, which is very high coloured, and what could be caught depoſits a copious ſediment; reſted better laſt night, owing to xv. drops of tinct. opii in his ſaline potion: is more reſtleſs this morning, and ſcreams with greater violence. Cont. medicam. et veſper, n. p. injiciend enema domeſtic.

*March* 5. Laſt night the enema was adminiſtered, which produced a copious evacuation; had a better night; was in a comatoſe ſtate when I viſited him, but upon diſturbing him, again relapſed into his former ſcreaming fit; he is nearly hoarſe, and is much debilitated. The bliſter upon his head is nearly dry.

dry. Applic. empl. veſicat. inter ſcapul. Cont. medicam. ut heri.

*March* 6. Reſted a little in the night, and is quieter to-day during the intervals of ſcreaming; does not moan as uſual; had another glyſter this morning, which operated like the former; ſkin rather moiſt; his nurſe thinks his breath ſmells very fœtid. Cont. medicam.

*March* 7. Bliſter diſcharges very much; ſeems to be ſenſible at intervals, and anſwers with a monoſyllable; the ſcreaming paroxyſm continues not ſo long or ſo violent; the pupils continue dilated, but not to ſo great a degree; has had a natural ſtool; urine in greater quantity, but ſtill involuntary; his breath continues fœtid, mouth a little ſore, tongue moiſt, which hitherto has been the contrary. Cont. med.

*March* 9. He neither ſcreams ſo often or violent as on the 7th, but is very petulant if diſturbed; his pulſe is very irritable; the bliſter ſtill diſcharges conſiderably; natural ſtool each day; ſleeps little in the night, and what he has is diſturbed. Cont. miſt. ſaline, calomel, et augeat dos. tinct. opii. ad gt. xx.

*March* 12. The ſcreaming has left him; the bliſter is in a healing ſtate, and he is mending as faſt as poſſible; a diarrhœa having occurred, I have diſcontinued the calomel.

*March* 16. Every complaint has now left him except debility; continues very petulant; the pupils are ſtill dilated beyond their natural ſtate, but contract on expoſure to light; his appetite will now take any thing given him. Hab. decoct. cinchonæ et omitt. op.

*April* 1. Has now recovered his uſual ſtrength, and there is no appearance of any relapſe.

During the whole of the time I attended this patient, or ſince his recovery, which was rapid, conſidering the violence of the diſeaſe, he has voided no worm; nor is there at preſent any ſymptom of their preſence.—I was led to preſent you with the above caſe, in conſequence of Mr. White's aſſerting, in your XIIth. Number, that he ſaw no good effects reſult from the uſe of mercury uncombined with digitalis; but can aſſure him I have proved its efficacy in ſeveral prior caſes, and the preſent recent one tends to convince me of its utility.

About four months ago a child, ſeven years old, who laboured under a complication of diſorders, with ſlight ſymptoms of hydrocephalus, after lingering ſome time, and taking a variety of remedies, at length the diſeaſe terminated fatally; upon examining the head, nearly two ounces of a limpid fluid was found in the ventricles, but no other unnatural appearance was perceptible.

